# miR-182-5p promotes hepatocellular carcinoma progression by repressing FOXO3a

**DOI:** 10.1186/s13045-018-0555-y

**Published:** 2018-01-24

**Authors:** Man-Qing Cao, A-Bin You, Xiao-Dong Zhu, Wei Zhang, Yuan-Yuan Zhang, Shi-Zhe Zhang, Ke-wei Zhang, Hao Cai, Wen-Kai Shi, Xiao-Long Li, Kang-Shuai Li, Dong-Mei Gao, De-Ning Ma, Bo-Gen Ye, Cheng-Hao Wang, Cheng-Dong Qin, Hui-Chuan Sun, Ti Zhang, Zhao-You Tang

**Affiliations:** 1 Department of Hepatobiliary Surgery, Liver Cancer Institute and Zhongshan Hospital, Fudan University, 180 Fenglin Road, Shanghai, 200032 China; 2 Department of Hepatobiliary Surgery, Tianjin Medical University Cancer Institute and Hospital, National Clinical Research Center for Cancer, Tianjin’s Clinical Research Center for Cancer, Key Laboratory of Cancer Prevention and Therapy, Tianjin, 300060 China; 3 Key laboratory of Carcinogenesis and Translational Research (Ministry of Education/Beijing), Division of Etiology, Peking University Cancer Hospital and Institute, Haidian District, Beijing, 100142 China; 4 Department of General Surgery, Xinhua Hospital, School of Medicine, Shanghai Jiao Tong University, Shanghai, 200092 China; 5 Department of Colorectal Cancer Surgery, Zhejiang Cancer Hospital, Zhejiang, Hangzhou 310022 China; 6 Department of Organ Transplantation, Changhai Hospital, The Second Military Medical University, Shanghai, 200433 China; 7 Department of Liver Surgery, Fudan University Shanghai Cancer Center, Cancer Hospital, Shanghai, 200032 China; 8 Department of Breast Cancer Surgery, Zhejiang Cancer Hospital, Zhejiang, Hangzhou 310022 China

**Keywords:** miR-182-5p, HCC, FOXO3a, Wnt signaling

## Abstract

**Background:**

High frequency of recurrence is the major cause of the poor outcomes for patients with hepatocellular carcinoma (HCC). microRNA (miR)-182-5p emerged as a high-priority miRNA in HCC and was found to be related to HCC metastasis. Whether the expression of miR-182-5p in tumor tissue correlated with early recurrence in HCC patients underwent curative surgery was unknown.

**Methods:**

Real-time PCR (RT-PCR) and in situ hybridization (ISH) were conducted to assess the expression of miR-182-5p in HCC cells and tissues. Cell Counting Kit-8 (CCK-8), transwell assays were performed to detected cells proliferation and migration ability. Flow cytometry assays were used to detect cell apoptosis rate, and xenograft model was employed to study miR-182-5p in HCC growth and lung metastasis. The target of miR-182-5p was validated with a dual-luciferase reporter assay and western blotting. Immunohistochemistry, immumoblotting, and immunoprecipitation were performed to test relative protein expression.

**Results:**

We showed that high expression of miR-182-5p in tumor tissues correlated with poor prognosis as well as early recurrence in HCC patients underwent curative surgery. miR-182-5p enhanced motility and invasive ability of HCC cells both in vitro and in vivo. miR-182-5p directly targets 3′-UTR of FOXO3a and repressed FOXO3a expression, activating AKT/FOXO3a pathway to promote HCC proliferation. Notably, miR-182-5p activated Wnt/β-catenin signaling by inhibiting the degradation of β-catenin and enhancing the interaction between β-catenin and TCF4 which was mediated by repressed FOXO3a.

**Conclusions:**

Consistently, miR-182-5p can be a potential predictor of early recurrence for HCC patients underwent curative surgery, and FOXO3a plays a key mediator in miR-182-5p induced HCC progression.

## Background

Hepatocellular carcinoma (HCC) is a prevalent malignancy that ranks the third leading cause of cancer mortality worldwide [1]. High frequencies of recurrence and metastasis are the major causes for the poor clinical outcomes of HCC patients. Increasing evidences have supported that miRNA deregulation is in correlation with HCC progression [2, 3]. By targeting the 3′-untranslated region (UTR) of the mRNA, miRNA leads to inhibition of targeted mRNA depending on total or partial complementarity [4, 5]. miR-182-5p, a member of the miR-183/96/182 cluster, emerged as a high-priority miRNA in HCC and has been proven to be related to various cancers. However, the function of miR-182-5p is complicated because it can be an oncogene or a tumor suppressor in the context of different cancers. miR-182-5p is identified as onco-miR in ovarian cancer [6], breast cancer [7], and melanoma [8] and acts as tumor suppressor in RCC [9, 10] and glioblastoma [11, 12]. In HCC, miR-182-5p contributes to HCC metastasis by targeting metastasis suppressor 1 (MTSS1) [13], and upregulated miR-182-5p increases drug resistance in cisplatin-treated HCC cells by regulating tumor protein 53-induced nuclear protein 1 (TP53INP1) [14]. In addition, increased miR-182-5p can be of diagnostic and prognostic value in HCC patients [15]. However, whether miR-182-5p was involved in early recurrence of HCC remained unknown. We here investigated the relationship between miR-182-5p and early recurrence of HCC patients underwent curative surgery and further explored the underlying mechanisms of miR-182-5p in promoting HCC progression.

FOXO3a, a member of Forkhead box O (FOXO) transcription factor family, mediates many genes through its transcriptional activity, with important roles in cell fate decisions and is also suggested to play a pivotal role as a tumor suppressor in a wide range of cancers [16–18]. FOXO3a is an important target of PI3K/Akt pathway. Activated AKT phosphorylates FOXO3a and leads to its cytoplasmic translocation and subsequently degradation [19]. In addition to AKT, there are other negative regulators of FOXO3a, such as serum and glucocorticoid-regulated kinase (SGK) [20, 21]. It has been reported that activation of the Wnt signaling pathway induced expression of SGK1 and lead to nuclear exclusion of FOXO3a [21], indicating that FOXO3a was under the regulation of Wnt/β-catenin signaling pathway. In contrast, FOXO3a was reported to inhibit the expression β-catenin by transactivating miR-34b/c in prostate cancer [22], and FOXO3a can directly bind to β-catenin and compete with T cell factor (TCF) for the interaction to β-catenin, thereby inhibiting β-catenin/TCF transcriptional activity [23].

In the present study, we demonstrated that miR-182-5p could be a potential predictor for early recurrence of HCC patients underwent curative surgery, and miR-182-5p acted as a promoter of HCC growth both in vitro and in vivo*.* Notably, we found that miR-182-5p activated Wnt signaling pathway by inhibiting the degradation of β-catenin and enhancing the interaction between β-catenin and TCF4, which was mediated by repressed FOXO3a. These results provide new insight into the mechanism of miR-182-5p in promoting HCC progression.

## Methods

### Cell culture and transfection

HEK293T was originally obtained from the American Type Culture Collection (ATCC). MHCC-97H and MHCC-97L are human hepatocellular carcinoma cell lines with high metastatic potential [24], obtained from Liver Cancer Institute. All cell lines were maintained in Dulbecco’s modified Eagle’s medium supplemented with 10% FBS, 10 U/mL penicillin, and 10 mg/mL streptomycin. Cells were grown in a humidified atmosphere at 37 °C at gas tensions of 20% O_2_/5% CO_2_ for normoxic incubation.

Plasmids used in the experiment:

miRNA inhibitor scrambled control: CmiR-AN0001-AM03, anti-miR-182-5p: HmiR-AN0239-AM03, miR-182-5p: HmiR0115-MR03, miR-NC: CmiR0001-MR03, and Lenti-Pac™ HIV Expression Packaging Kit (Cat.No. HPK-LvTR-20) were all purchased from the GeneCopoeia.

### Cellular proliferation assay and transwell assay

For the cell proliferation assay, 5 × 10^3^ cells were plated in 96-well plates. Cell growth was determined by using CCK8 assay. After transfection, MHCC-97H and MHCC-97L cells were suspended in 100 μL serum-free medium and placed in the upper chambers of the transwell and incubated at 37 °C for 72 h for the invasion assay. The cells that penetrated the matrigel-coated filters were counted at a magnification of × 200 in eight randomly selected fields, and the mean number of cells per field was recorded.

### Immunoprecipitation

Whole-cell extracts were prepared in immunoprecipitation (IP) lysis buffer. The extracts were incubated overnight at 4 °C with 4 μg antibody and for an additional 2 h with protein A/G agarose beads. Beads were then washed three times with the lysis buffer, and the beads with the immunoprecipitates were resuspended in sodium dodecyl sulfate polyacrylamide gel electrophoresis (SDS–PAGE) sample buffer and heated at 95 °C for 10 min. Subsequently, the supernatants were analyzed by SDS-PAGE followed by western blot.

### Western blotting

Western blot analyses were performed as previously described [25, 26].

AKT #4685 CST; p-AKT Ser473 #4060 CST; Bcl-2 #15071; Bcl-xl #2764 CST; Cyclin D1 #2922 CST; SGK #12103 CST; β-actin #3700 CST; Wnt3a #2721 CST; Wnt/β-catenin Activated targets antibody Sampler Kit #8655 CST; Forkhead Signaling Antibody Sampler Kit #9946 CST.

### RNA preparation and real time-PCR (RT-PCR)

Total RNA including miRNAs was extracted from cells using TRIzol reagent (Invitrogen) following the manufacturer’s protocol. For analysis of miR-182-5p expression, reverse transcription and stem-loop RT-PCR were performed using the TaqMan MicroRNA assays (Applied Biosystems, Foster City, CA, USA) and amplified by TaqMan Universal PCR Master Mix (Applied Biosystems). U6 snRNA was probed as a loading control.

### In situ hybridization (ISH)

ISH was used to detect miR-182-5p in tissue microarrays using digoxigenin-labeled sense and antisense miR-182-5p probes (Exiqon, 610341-360, Denmark). The slides were de-paraffined and rehydrated before incubation with Proteinase K at 37 °C for 40 min, then wash the slides three times with PBS for 15 min. After incubation with 5× SSC solution at room temperature for 15 min, miR-182-5p probes were added for hybridization at 50 °C for 1 h. Next, the slides were washed with graded-diluted SSC solutions at 50 °C for 30 min, followed by incubation with an antibody against digoxigenin (1:1000, Roche, Mannheim, Germany) at 4 °C overnight. Finally, hybridization signals were visualized by NBT/BCIP (Sigma), and the reaction was stopped by washing with water for 5 min. At last, slides were counterstained with nuclear fast red for 1 min and then mounted using an aqueous solution.

### Dual-luciferase reporter assay

The 3′-UTR segments of FOXO3a including the wild type or the mutant type of miR-182-5p binding sites were cloned into the downstream of the luciferase reporter, the pmirGLO Dual-Luciferase miRNA Target Expression Vector (Promega, Madison, USA), between the SacI and SalI sites and verified by sequencing. HEK 293 T cells were plated into 24-well plates and transfected with 50 nM miR-182-5p or NC and 100 ng of the luciferase vector (pmirGLO). Cells were harvested 48 h after the transfection. The relative luciferase activity was measured by the Dual-Glo luciferase assay kit (Promega).

### Human samples

This study was approved by the clinical research ethics committee of the Shanghai Zhongshan Hospital of Fudan University. The tumor specimens for tissue microarrays were obtained from 119 patients who underwent surgery during July 2014 to May 2015 at the Shanghai Zhongshan Hospital of Fudan University. The disease-free survival was calculated from the date of resection to the date of tumor recurrence.

### Tumor xenografts in nude mice

The orthotopic HCC implant tumor model in nude mice was performed as previously described [25, 26]. All surgical procedures and care administered to the animals were in accordance with the institutional ethics guidelines.

### Statistical analysis

Immunohistochemistry (IHC) data were analyzed using a χ^2^ test. A two-tailed *t* test was used to compare the means between two sets, and a one-way analysis of variance was used to compare the means among three groups. By the Kaplan-Meier method and the log-rank test, survival curve analysis was performed to study the role of miR-182-5p in HCC progression. The data were analyzed with SPSS software version 19.0 (SPSS Inc., Chicago, IL, USA). *P* < 0.05 (two-sided) was considered statistically significant.

## Results

### miR-182-5p is upregulated in HCC cell lines and human HCC tissues, and its high expression predicts poor prognosis and early recurrence of HCC

**Fig. 1:**
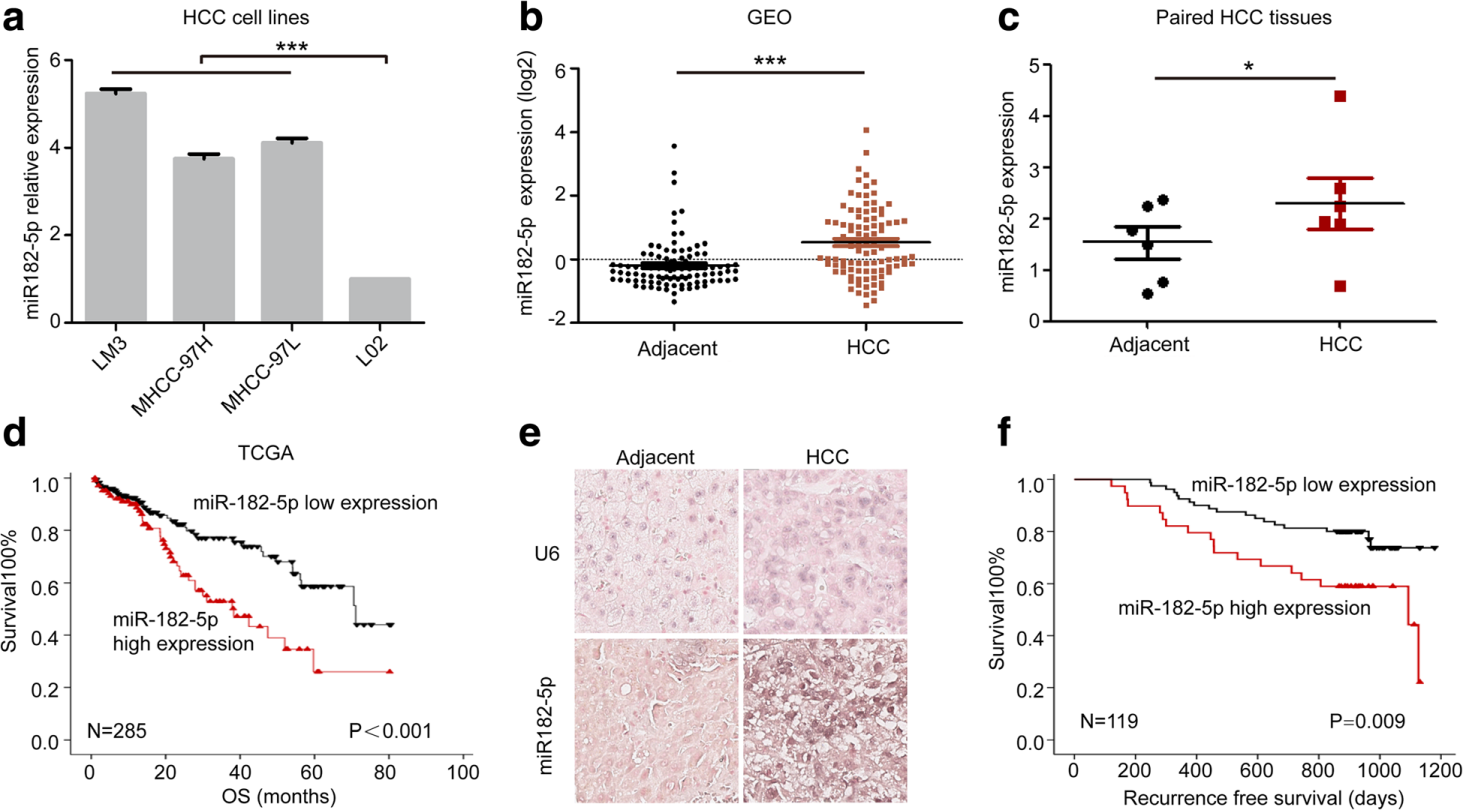
miR182-5p can be regarded as predictor for early recurrence of HCC. **a** Relative expression of miR-182-5p in different HCC cell lines. **b** Relative expression of miR-182-5p in HCC and adjacent tissues of GEO database (GSE22058). **c** Relative miR-182-5p expression in paired HCC tissues and adjacent tissues. **d** The relationship between expression of miR-182-5p and overall survival of HCC patients in TCGA database. **e** ISH staining of U6 and miR-182-5p in HCC and adjacent tissues. **f** The relationship between expression of miR-182-5p and recurrence-free survival of HCC patients in Zhongshan hospital. (*P < 0.05, **P < 0.01, ***P < 0.001)

We firstly examined the expression level of miR-182-5p in several HCC cell lines and the normal liver cell line L02. miR-182-5p expressed relatively higher in HCC cell lines than in L02 cells (Fig. 1a). By using GEO database (GSE22058) [27], we compared the expression of miR-182-5p in HCC and adjacent liver tissues. The mean level of miR-182-5p was higher in HCC tissues than that in adjacent liver tissues (Fig. 1b). We further analyzed the miR-182-5p expression in six paired HCC and adjacent tissues of patients in Liver Cancer Institute and Zhongshan Hospital, Fudan University; the mean level of miR-182-5p was also higher in HCC tissues than that in adjacent liver tissues (Fig. 1c). By employing the TCGA database, we further analyze the relationship between miR-182-5p and HCC prognosis. The results showed that patients with high miR-182-5p expression exhibited significantly shorter overall survival than patients with low miR-182-5p expression (*P* < 0.001) (Fig. 1d). ISH staining was used to detect the miR-182-5p expression in tissue microarray containing 119 human HCC patients with miR-182-5p probes (Fig. 1e). Patients with high miR-182-5p expression showed shorter recurrence-free survival than patients with low miR-182-5p expression (Fig. 1f). (*P* = 0.009, Liver Cancer Institute and Zhongshan Hospital, Fudan University).

We further analyzed the correlation between miR-182-5p expression and clinicopathological parameters, and only AFP levels showed significant statistical difference (Table 1). Univariate analyses of prognostic factors for recurrence-free survivals showed that staining intensity of miR-182-5p, micro vein invasion (MVI), and AFP level had significant difference (Table 2). Multivariate analyses of prognostic factors for recurrence-free survivals showed that only the miR-182-5p staining intensity had the difference of statistics (Table 3). Taken together, these results implied that miR-182-5p could be a predictor for early recurrence of HCC.

**Table 1:** Correlation between miR-182-5p expression and HCC clinicopathological parameters

Parameters	*n*	miR-182-5p		*P* value
		Low (*n* = 80)	High (*n* = 39)	
Gender				
Male	100	66	34	0.602
Female	19	14	5	
Age (years)				
< 50	30	18	12	0.372
≥ 50	89	62	27	
Cirrhosis				
Absence	27	22	5	0.102
Presence	92	58	34	
Tumor number				
Single	96	66	30	0.471
Multiple	23	14	9	
Tumor size(cm)				
< 5 cm	102	70	32	0.418
≥ 5	17	10	7	
Micro vein invasion				
Absence	77	54	23	0.416
Presence	42	26	16	
HBV infection				
Absence	16	13	3	0.259
Presence	103	67	36	
AFP				
< 20	54	42	12	0.031*
≥ 20	65	38	27	

*AFP* alpha-fetoprotein

*P < 0.05

**Table 2:** Univariate analyses of prognostic factors for recurrence-free survivals

Parameters	*n*	Chi-square	*P* value
Gender		0.084	0.772
Male	100		
Female	19		
Age		0.009	0.924
< 50	30		
≥ 50	89		
Cirrhosis		0.732	0.392
Absence	27		
Presence	92		
Staining intensity		6.728	0.009*
Low	80		
High	39		
Tumor number		1.177	0.278
Single	96		
Multiple	23		
Tumor size		0.000	0.986
< 5 cm	102		
≥ 5	17		
Micro vein invasion		4.809	0.028*
Absence	77		
Presence	42		
HBV infection		0.861	0.353
Absence	16		
Presence	103		
AFP		5.859	0.015*
< 20	54		
≥ 20	65		

*P < 0.05

**Table 3:** Multivariate analyses of prognostic factors for recurrence-free survivals

Parameters	B	SE	*P* value	Exp(B)
Staining intensity	− 0.713	0.341	0.036*	0.490
Micro vein invasion	0.554	0.345	0.108	1.740
AFP	0.592	0.382	0.121	1.808

*P < 0.05

### miR-182-5p promotes HCC growth both in vitro and in vivo

**Fig. 2:**
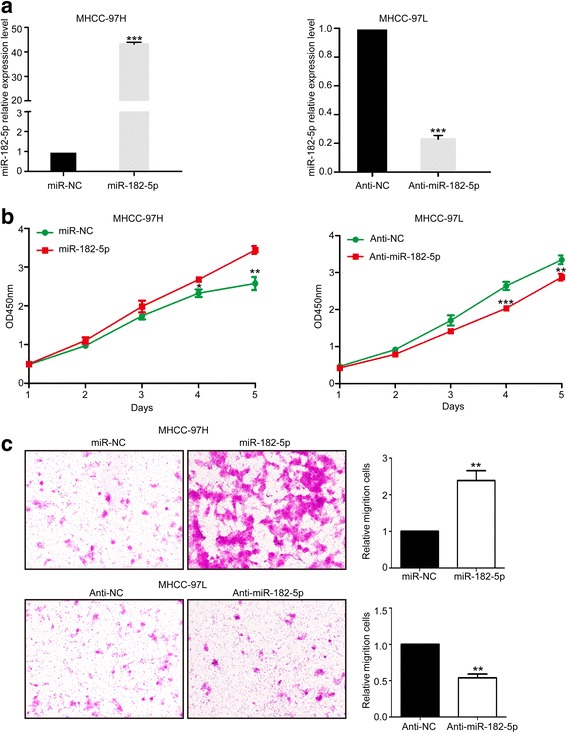
Effects of miR182-5p in HCC proliferation and invasion. **a** Relative miR-182-5p expression in HCC cells that overexpressed miR-182-5p in MHCC-97H cells and knockdown miR-182-5p in MHCC-97L cells. **b** CCK8 assays of miR-NC cells and miR-182-5p-overexpression cells of MHCC-97H, and CCK8 assays of Anti-NC cells and miR-182-5p-inhibition cells of MHCC-97L cells. **c** Transwell assays of miR-NC cells and miR-182-5p-overexpression cells of MHCC-97H, and transwell assays of anti-NC cells and miR-182-5p-inhibition cells of MHCC-97L cells. (*P < 0.05, **P < 0.01, ***P < 0.001)

**Fig. 3:**
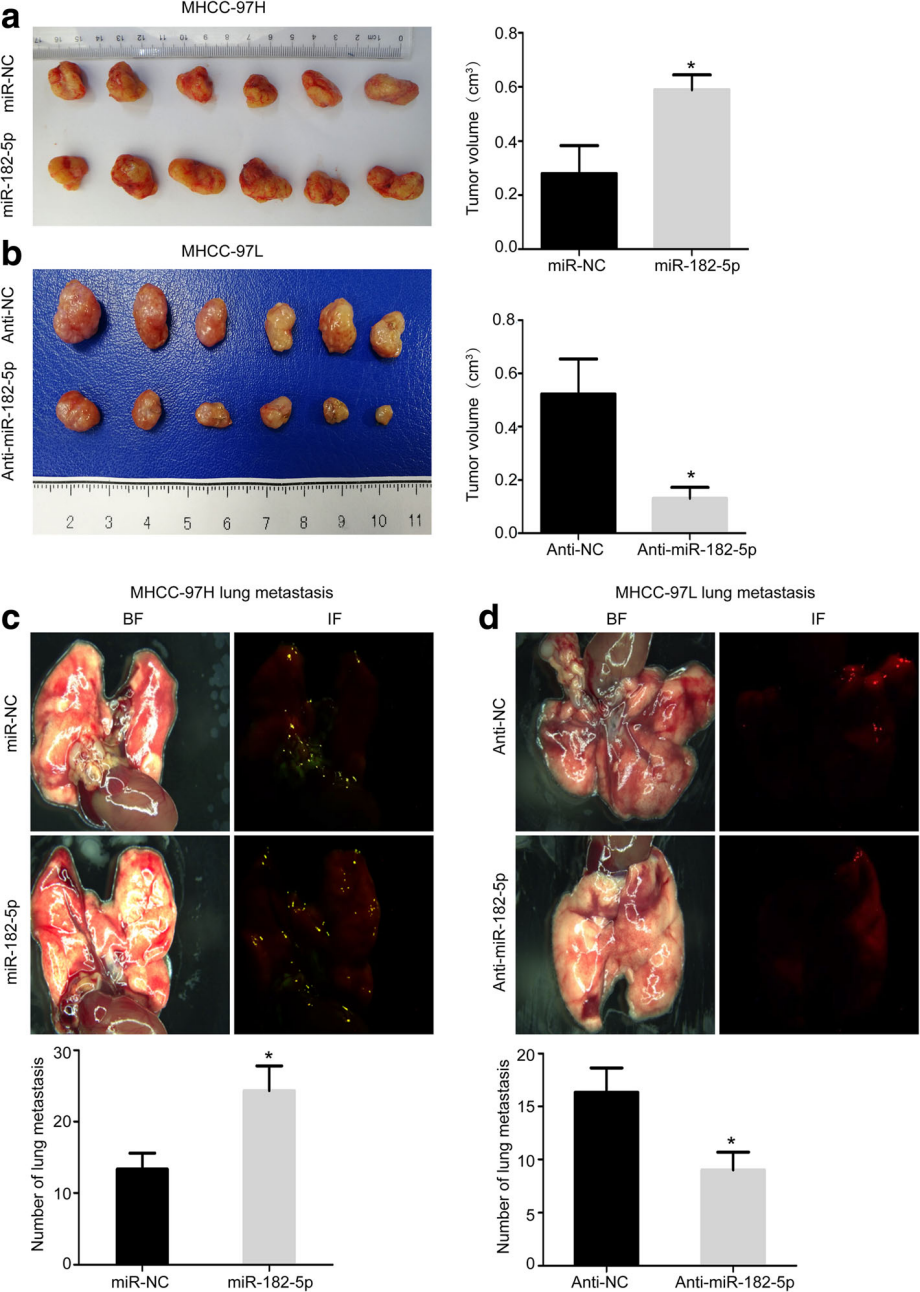
The role of miR-182-5p in HCC growth and lung metastasis. **a** Orthotopic transplantation tumor formation with miR-NC and miR-182-5p overexpression in MHCC-97H cells, and tumor size analysis of the miR-NC and miR-182-5p overexpression groups. **b** Orthotopic transplantation tumor formation with anti-NC and miR-182-5p inhibition in MHCC-97L cells, and tumor size analysis of the anti-NC and miR-182-5p inhibition groups. **c** The images of lung metastasis of miR-NC and miR-182-5p-overexpression groups by stereo fluorescence microscope and lung metastasis analysis of miR-NC and miR-182-5p-overexpression groups. **d** The images of lung metastasis of anti-NC and miR-182-5p-inhibiton groups by stereo fluorescence microscope, and lung metastasis analysis of anti-NC and miR-182-5p-inhibition groups. (*P < 0.05, **P < 0.01, ***P < 0.001)

To investigate the effect of miR-182-5p on HCC, we transduced lentivirus to stably overexpress miR-182-5p expression in MHCC-97H cells and knockdown miR-182-5p in MHCC-97L cells (Fig. 2a). CCK8 assay showed that overexpression of miR-182-5p promoted HCC growth and clone formation ability in comparison with the control group. In contrast, knockdown miR-182-5p suppressed the growth of HCC cells (Fig. 2b). Based on the transwell assay, miR-182-5p overexpression significantly enhanced the invasion abilities of HCC cell, and knockdown miR-182-5p weakened the invasive ability of HCC cells (Fig. 2c). To further assess the function of miR-182-5p in tumor progression and metastasis, we used orthotropic transplantation model of HCC in nude mice. We evaluated the tumor sizes and lung metastases of these liver orthotropic models with different transfected cells. The tumor sizes and lung metastases of miR-182-5p overexpression group were greatly increased compared to the control group (Fig. 3a, c). The miR-182-5p knockdown group obviously delayed tumor sizes and number of lung metastases (Fig. 3b, d). Our data demonstrated that miR-182-5p played a role in the progression of HCC.

### miR-182-5p binds to 3′-UTR of FOXO3a

**Fig. 4:**
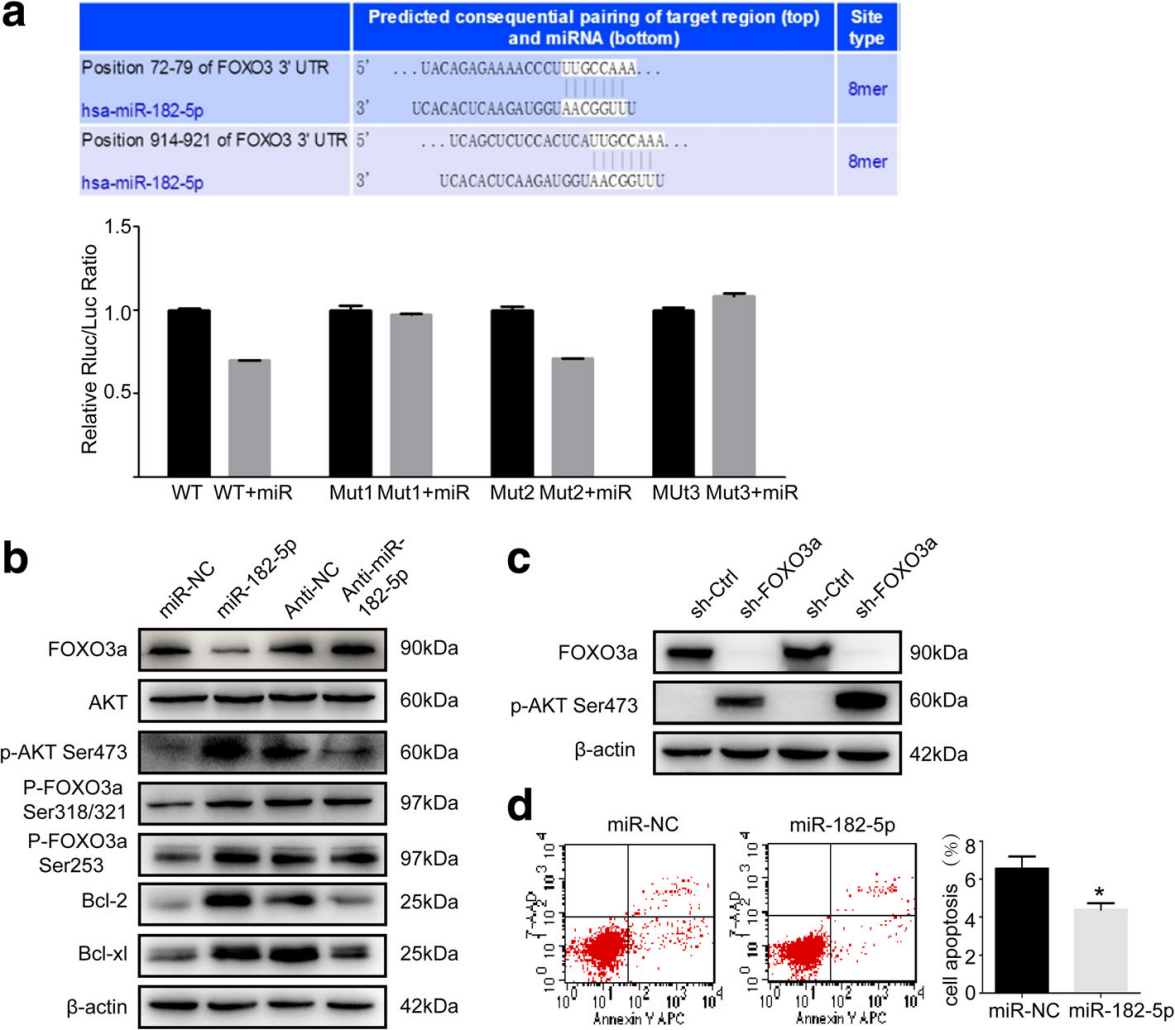
miR-182-5p targets FOXO3a to activate AKT pathway. **a** Predicted binding sites of 3′-UTR of FOXO3a to miR-182-5p, and the relative Rluc/Luc ratio of dual-luciferase reporter assay in different groups. **b** Western blot analysis of AKT/FOXO3a signaling-related proteins in control group, overexpression and knockdown miR-182-5p group of HCC cells. **c** Western blot analysis of p-AKT Ser473 expression after FOXO3a knockdown. **d** FASC analysis of cell apoptosis rate in miR-NC and miR-182-5p overexpression cells

To identify specific gene targets of miR-182-5p through which it might promote oncogenic behavior in vitro and in vivo, the public algorithms (TargetScan, Pictar, miRANDA, miRWalk) were used. Among hundreds of predicted targets, FOXO3a was chosen not only for the reason that it was identified as an anti-oncogene but also because of its relatively good scores of predicted binding sites (Fig. 4a).

To verify the interaction between miR-182-5p and FOXO3a in HCC, luciferase reporter assay was performed. There are two binding site 72–79 site and 914–921 site of 3′-UTR of FOXO3a that could possibly bind with miR-182-5p (Fig. 4a). The wild-type vectors, two mutant 3′-UTR of FOXO3a mRNA, and a double mutant vector were built to identify which sequence was responsible for binding to miR-182-5p. The luciferase activity of the wild-type vector group was significantly inhibited by miR-182-5p. The luciferase activity in the FOXO3a mutant2 (M2) group was also inhibited, but luciferase activity of FOXO3a mutant1 (M1) group and M1 + M2 group was not suppressed by miR-182-5p mimics (Fig. 4a). These results indicated that miR-182-5p might inhibit FOXO3a expression by binding to 72–79 site but not the 914–921 site of 3′-UTR of FOXO3a.

### miR-182-5p promotes HCC cells proliferation by activating AKT

The underlying mechanisms of miR-182-5p on tumor growth were further explored. We observed a significant increase in AKT phosphorylation following miR-182-5p overexpression (Fig. 4b). In contrast, AKT phosphorylation was inhibited by knockdown of miR-182-5p (Fig. 4b). Multiple studies have identified that FOXO3a was a downstream target of AKT [28]. To confirm AKT was activated, we further detected the expression of phosphorylation of FOXO3a in miR-182-5p overexpression and knockdown cells. Two sites of FOXO3a phosphorylation were both upregulated after miR-182-5p overexpression (Fig. 4b). Moreover, Bcl-2 and Bcl-xl, the downstream target of AKT, were both upregulated following miR-182-5p overexpression (Fig. 4b). In order to explore FOXO3a was involved in increased phosphorylated AKT by miR-182-5p. We next established a stably FOXO3a knockdown cell line in MHCC-97H cells and measure the phosphorylation of AKT expression. The results showed that FOXO3a knockdown could greatly increase the expression of phosphorylation AKT, forming a positive feedback loop (Fig. 4c). As Bcl-2 and Bcl-xl were important anti-apoptosis proteins, we further detected the rate of apoptosis in miR-182-5p overexpression cells by fluorescence-activated cell sorting (FACS). Forced expression of miR-182-5p remarkably decreased the apoptosis rate (Fig. 4d). Thus, we proposed that overexpression of miR-182-5p activated AKT/FOXO3a signaling by repressing FOXO3a to promote proliferation of HCC cells.

### miR-182-5p promotes HCC metastasis by activating Wnt signaling

**Fig. 5:**
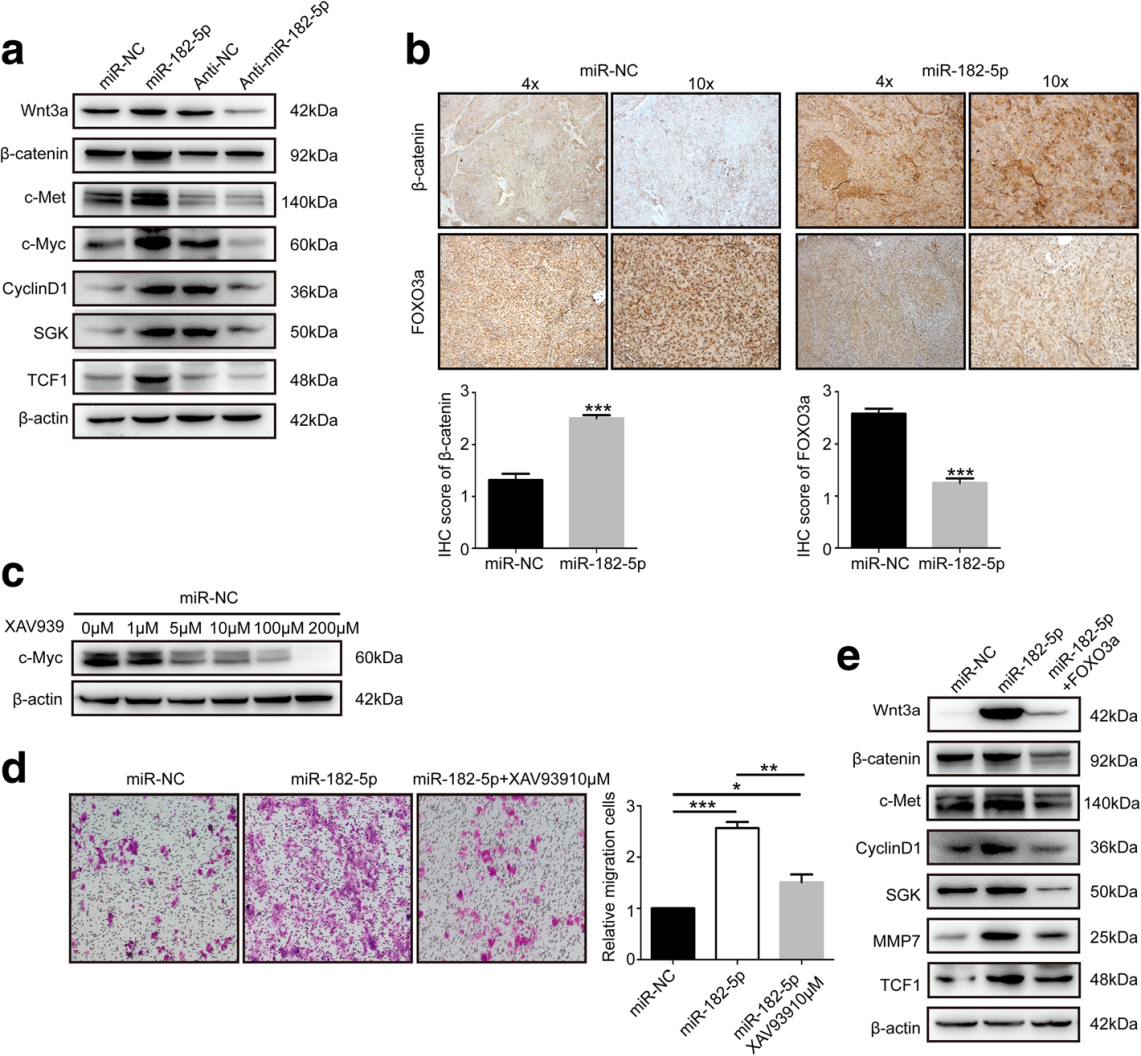
FOXO3a exerts an important mediator in miR-182-5p induced Wnt signaling activation. **a** Western blot analysis of Wnt signaling pathway related proteins following miR-182-5p overexpression and knockdown. **b** IHC staining of FOXO3a and β-catenin in orthotopic tumor tissues of negative control and overexpression of miR-182-5p. **c** Western blot analysis of c-Myc in miR-NC cells treated with different concentration of XAV939 (0, 1, 5, 10, 100, and 200 μM). **d** Transwell assay of HCC cells that transfected with miR-NC, overexpression of miR-182-5p and miR-182-5p overexpression cells that further treated with 10 μM XAV939. **e** Western blot analysis of Wnt signaling pathway-related proteins in miR-NC cells, overexpression of miR-182-5p cells and miR-182-5p overexpression cells further overexpress FOXO3a

Our results showed that forced miR-182-5p expression increased Wnt3a expression. Further, we found that the downstream targets of Wnt signaling pathway were activated (Fig. 5a). By using IHC staining, we detected the expression of FOXO3a and β-catenin in orthotopic transplantation mice model tissues and showed that miR-182-5p remarkably suppressed FOXO3a whereas promoted β-catenin expression in HCC mouse model (Fig. 5b). These results supported that Wnt signal pathway was activated by miR-182-5p.

In order to prove that Wnt signaling was involved in the enhanced metastasis ability by miR-182-5p, we used XAV939, a specific inhibitor of Wnt signaling pathway, to inhibit Wnt signal. Different concentration, 0,1,5,10,100, and 200 μM, were used to treat HCC cells. After 12 h, we detected the c-Myc expression, an important effector of Wnt signaling pathway and found that c-Myc gradually decreased along with the increasing concentration of XAV939 (Fig. 5c). We choose 5 μM as a proper concentration for the subsequent treatment. Next, we employed the transwell assay to analyze the invasive ability of the control group, overexpression miR-182-5p group, and miR-182-5p overexpression with XAV939 group. The results showed that Wnt pathway inhibitor could restrain the invasiveness induced by miR-182-5p (Fig. 5d).

As FOXO3a has been proved to be related to Wnt signal regulation, we hypothesized that FOXO3a might play a role in the activation of Wnt signal pathway by miR-182-5p. We further overexpressed FOXO3a in miR-182-5p overexpression cells and detected the downstream targets of Wnt signaling pathway. The expression of Wnt3a and its downstream targets were suppressed compared to the miR-182-5p overexpressing cells (Fig. 5e), which implied that miR-182-5p activated the Wnt signaling pathway by modulating the expression of FOXO3a.

### miR-182-5p activates Wnt signaling pathway through inhibiting β-catenin degradation and enhancing β-catenin/TCF4 interaction via FOXO3a

**Fig. 6:**
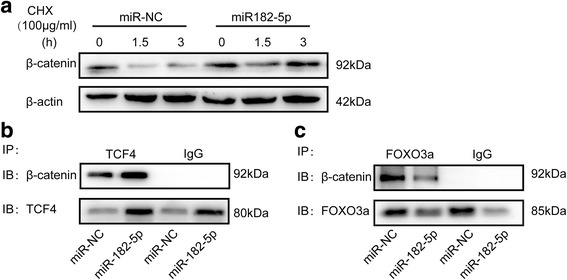
miR-182-5p inhibits β-catenin degradation and enhances the binding of β-catenin/TCF4. **a** Western blot analysis of β-catenin expression in miR-NC and miR-182-5p overexpression cells that treated with 100 μg/mL CHX at 0, 1.5, and 3 h. **b** Endogenous TCF4 was immunoprecipitated, and binding of β-catenin to TCF4 was analyzed by immunoblotting for β-catenin. **c** Endogenous FOXO3a was immunoprecipitated, and binding of β-catenin to FOXO3a was analyzed by immunoblotting for β-catenin

β-catenin is a key inducer for the Wnt pathway, where in unstimulated cells, β-catenin proteins in the cytoplasm are phosphorylated and degraded by the proteasome [29, 30]. Following Wnt signaling pathway activation, β-catenin was accumulated and localized into the nucleus to activate TCF/LEF transcription factors to initiate the expression of Wnt signaling target genes such as c-MYC and cyclin D1 [31]. To investigate how miR-182-5p activates Wnt signal pathway, we hypothesized that miR-182-5p overexpression might suppress β-catenin degradation. We therefore used protein synthesis inhibitor cycloheximide (CHX) to treat the control cells and miR-182-5p overexpression cells. After 1.5 and 3 h, the expression of β-catenin was measured by western blot. The degradation of β-catenin in the control cells was obviously faster than the miR-182-5p overexpression cells (Fig. 6a). Increasing evidences proved that FOXO3a can directly bind to β-catenin and competes with TCF for interaction with β-catenin [22, 23]. By using the co-IP assays, we found that miR182-5p enhanced the interaction between β-catenin and TCF4, meanwhile, the interaction between β-catenin and FOXO3a was impaired (Fig. 6b). In conclusion, these results demonstrated that miR-182-5p activated Wnt signaling pathway by stabilization β-catenin and promoting β-catenin/TCF4 activity via inhibiting FOXO3a.

## Discussion

Metastasis and recurrence are the main problems that limiting the 5 years’ survival rate of HCC. In HCC patients, high-serum VEGF has been shown to associate with tumor recurrence, metastasis, and poor survival [32]. In addition to targeted drugs for VEGF in HCC, non-invasive detection of hydrodynamic changes in the field of industry has made a lot of progress [33, 34], which might be applied in the near future in the field of medicine.

Our results showed that miR-182-5p was overexpressed in both HCC cell lines and HCC tissues. Moreover, miR-182-5p was related to poor prognosis and early recurrence in HCC. By univariate and multivariate analyses, we found that the expression of miR-182-5p could be regarded a potential predictor for early recurrence of HCC patients under curative surgery.

miR-183, miR-182, and miR-96 act as regulators of FOXO expression in various cancer types [35, 36]. MiR-182-5p has been reported to promote melanoma metastasis by repressing FOXO3 [8]. However, the specific binding target site in the 3′-UTR was not showed. Here, we exhibited that miR-182-5p suppressed FOXO3a expression by binding to the 72–79 site, but not 914–921 site in the 3′-UTR of FOXO3a.

Overexpression of miR-182-5p induces G1-phase arrest via inhibition of AKT/FOXO3a signaling in RCC [9]. However, we proved that miR-182-5p promoted AKT phosphorylation resulting in inactivation of FOXO3a in HCC. Moreover, knockdown of FOXO3a could further activate AKT, indicating a positive feedback loop between AKT and FOXO3a. The apoptosis inhibiting protein Bcl-2 and Bcl-xl, downstream target of AKT, were upregulated by overexpression of miR-182-5p. Taken together, miR-182-5p promoted cell proliferation by repressing FOXO3a, subsequently activating AKT/FOXO3a pathway.

It has been shown that Wnt/β-catenin activated miR-183/96/182 expression in hepatocellular carcinoma and promoting cell invasion [37]. Furthermore, β-catenin enhances expression of primary and mature miR-96, miR-182, and miR-183 [38]. We proved that miR-182-5p activated Wnt signaling by inhibiting β-catenin degradation and promoting β-catenin/TCF4 interaction, forming a positive feedback loop between Wnt signaling and miR-182-5p.

Wnt/β-catenin signaling pathway plays a critical role in the proliferation and cell cycle regulation of HCC cells [39]. When Wnt signaling is activated, β-catenin accumulates in the nucleus interacting with the T cell factor/lymphoid enhancer-binding factor (TCF/LEF) to transcriptionally activate downstream gene expression, such as cyclin D and c-Myc which exert promoting role in cell cycle [31, 40]. β-catenin directly binds to FOXO and enhances FOXO transcriptional activity which is particularly important under condition of oxidative stress [41]. FOXO3a competes with TCF for interaction with β-catenin, thereby inhibiting TCF transcriptional activity [23]. Thus, β-catenin appears function as a switch that determining whether a cell chooses TCF or FOXO3a in response to stimuli.

We showed that miR-182-5p activated Wnt signaling by inhibiting β-catenin degradation, and miR-182-5p negatively regulate FOXO3a, resulting in the enhancement of β-catenin/TCF interaction, increasing the expression of β-catenin targeted genes. As a downstream factor of Wnt signaling, SGK1 preferentially phosphorylates Ser315, and AKT mediates the phosphorylation of Ser253 of FOXO3a [42]. SGK1 negatively regulates the transcription factor FOXO3a via phosphorylation and exclusion from the nucleus, leading to FOXO3a degradation [21]. When Wnt signal was activated by miR-182-5p, SGK was upregulated and lead to the phosphorylation of FOXO3a. As a result, the inactivation of FOXO3a might further promote Wnt signal, forming a positive feedback loop.

## Conclusions

In conclusion, our results proved that miR-182-5p acted as a promoter in HCC and could be regarded as predictor for early recurrence of HCC patients underwent curative surgery. miR-182-5p negatively modulate FOXO3a by targeting 3′-UTR of mRNA in 72–79 site. We also proposed that FOXO3a played a key mediator in miR-182-5p induced malignant behaviors of HCC, which may provide new insight into the mechanism of miR-182-5p in HCC progression.

## Abbreviations

ATCC: American Type Culture Collection
CCK-8: Cell Counting Kit-8
FACS: Fluorescence-activated cell sorting
FOXO: Forkhead box O
HCC: Hepotacellular carcinoma
IHC: Immunohistochemistry
IP: Immunoprecipitation
LEF: Lymphoid enhancer-binding factor
M1: Mutant1
M2: Mutant2
miR: MicroRNA
MTSS1: Metastasis suppressor 1
RT-PCR: Real time-PCR
SDS–PAGE: Sodium dodecyl sulfate polyacrylamide gel electrophoresis
SGK: Serum and glucocorticoid-regulated kinase
TCF: T cell factor
TP53INP1: Tumor protein 53-induced nuclear protein 1
UTR: Untranslated region
VEGF: Vascular endothelial growth factor
