# Correction to: miR-182-5p promotes hepatocellular carcinoma progression by repressing FOXO3a

**DOI:** 10.1186/s13045-018-0599-z

**Published:** 2018-04-18

**Authors:** Man-Qing Cao, A-Bin You, Xiao-Dong Zhu, Wei Zhang, Yuan-Yuan Zhang, Shi-Zhe Zhang, Kei-wei Zhang, Hao Cai, Wen-Kai Shi, Xiao-Long Li, Kang-Shuai Li, Dong-Mei Gao, De-Ning Ma, Bo-Gen Ye, Cheng-Hao Wang, Cheng-Dong Qin, Hui-Chan Sun, Ti Zhang, Zhao-You Tang

**Affiliations:** 1 Department of Hepatobiliary Surgery, Liver Cancer Institute and Zhongshan Hospital, Fudan University, 180 Fenglin Road, Shanghai, 200032 China; 2 Department of Hepatobiliary Surgery, Tianjin Medical University Cancer Institute and Hospital, National Clinical Research Center for Cancer, Tianjin’s Clinical Research Center for Cancer, Key Laboratory of Cancer Prevention and Therapy, Tianjin, 300060 China; 3 Key laboratory of Carcinogenesis and Translational Research (Ministry of Education/Beijing), Division of Etiology, Peking University Cancer Hospital and Institute, Haidian District, Beijing, 100142 China; 4 Department of General Surgery, Xinhua Hospital, School of Medicine, Shanghai Jiao Tong University, Shanghai, 200092 China; 5 Department of Colorectal Cancer Surgery, Zhejiang Cancer Hospital, Hangzhou, 310022 Zhejiang China; 6 Department of Organ Transplantation, Changhai Hospital, The Second Military Medical University, Shanghai, 200433 China; 7 Department of Liver Surgery, Fudan University Shanghai Cancer Center, Cancer Hospital, Shanghai, 200032 China; 8 Department of Breast Cancer Surgery, Zhejiang Cancer Hospital, Hangzhou, 310022 Zhejiang China

The original article [1] contains an error in Fig. 5a whereby the Western blot bands representing CyclinD1 have mistakenly been duplicated over the Western blot bands intended to represent SGK.

**Fig. 5:**
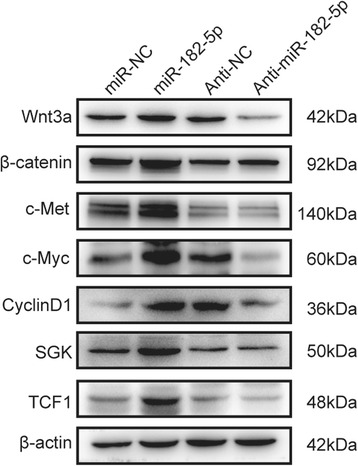
FOXO3a exerts an important mediator in miR-182-5p induced Wnt signaling activation. **a** Western blot analysis of Wnt signaling pathway related proteins following miR-182-5p overexpression and knockdown. **b** IHC staining of FOXO3a and β-catenin in orthotopic tumor tissues of negative control and overexpression of miR-182-5p. **c** Western blot analysis of c-Myc in miR-NC cells treated with different concentration of XAV939 (0, 1, 5, 10, 100, and 200 μM). **d** Transwell assay of HCC cells that transfected with miR-NC, overexpression of miR-182-5p and miR-182-5p overexpression cells that further treated with 10 μM XAV939. **e** Western blot analysis of Wnt signaling pathway-related proteins in miR-NC cells, overexpression of miR-182-5p cells and miR-182-5p overexpression cells further overexpress FOXO3a

As such, the correct version of Fig. 5a with the correct representation of the Western blots of SGK can be seen ahead.
